# Predicting coal workers’ pneumoconiosis trends: Leveraging historical data with the GARCH model in a Chinese Miner Cohort

**DOI:** 10.1097/MD.0000000000037237

**Published:** 2024-02-16

**Authors:** Peng Sun, Bosheng Wang, Hengdong Zhang, Ming Xu, Lei Han, Baoli Zhu

**Affiliations:** a Jiangsu Provincial Center for Disease Prevention and Control, Nanjing, Jiangsu, China; b Disease Prevention and Control Center of Liyang City, Changzhou, Jiangsu, China; c Key Laboratory of Environmental Medicine Engineering of Ministry of Education, School of Public Health, Southeast University, Nanjing, Jiangsu, China; d Center for Global Health, Nanjing Medical University, Nanjing, China; e Jiangsu Province Engineering Research Center of PublicHealth Emergency, Nanjing, Jiangsu, China.

**Keywords:** ARIMA, coal worker’s pneumoconiosis, GARCH, prediction

## Abstract

Coal workers’ pneumoconiosis (CWP) is one of the most common and severe occupational diseases worldwide. The main risk factor of CWP is exposure to respirable mine dust. Prediction theory was widely applied in the prediction of the epidemic. Here, it was used to identify the characteristics of CWP today and the incidence trends of CWP in the future. Eight thousand nine hundred twenty-eight coal workers from a state-owned coal mine were included during the observation period from 1963 to 2014. In observations, the dust concentration gradually decreased over time, and the incidence of tunnels and mine, transportation, and assistance workers showed an overall downward trend. We choose a better prediction model by comparing the prediction effect of the Auto Regression Integrate Moving Average model and Generalized Autoregressive Conditional Heteroskedasticity model. Compared with the Auto Regression Integrate Moving Average model, the Generalized Autoregressive Conditional Heteroskedasticity model has a better prediction effect. Furthermore, the status quo and future trend of coal miners’ CWP are still at a high level.

## 1. Introduction

Coal workers’ pneumoconiosis (CWP) is one of the most common and severe occupational diseases caused by prolonged inhalation of coal dust containing a concentration of free crystalline silica.^[1,2]^ CWP is still a global occupational health problem, especially in developing countries.^[1,3,4]^ Inhalation of coal mine dust induces chronic inflammation and progressive fibrosis, eventually leading to respiratory failure and death.^[5]^ Unfortunately, it is an irreversible progression, and no effective treatment exists.^[6]^

China is one of the countries with the most occupational pneumoconiosis in the world.^[7–9]^ It accounted for 85% to 90% of all reported occupational diseases annually in China during the last decade.^[10]^ Searching for the epidemiological characteristics of CWP in the past and present will help us to predict the future morbidity trend. Further, find out the long-term changes in CWP incidence and the factors that influence the morbidity in this relatively stable population of coal mine workers.^[11–13]^ In addition, disease prediction is a crucial step in transforming from passive prevention to active prevention, promoting the overall levels of prevention and control of occupational diseases and other public health issues.^[11]^

Here, a historical cohort study was performed to explore the epidemiological situation of CWP. All the workers exposed to coal mine dust are from 1 state-owned mine in eastern China with a mine history of 60 years, which ensures that the subjects are homogeneous. Our primary purpose is to predict the future incidence rates of CWP and provide a piece of reliable evidence for the government to develop more effective occupational health strategies.

## 2. Methods

### 2.1. Patient and public involvement

Patients and/or the public were not involved in this research’s design, conduct, reporting, or dissemination plans.

### 2.2. Study population

A total of 8928 coal miners with a history of mine dust exposure were included in the study, among which 495 have been diagnosed with CWP. All the participants are genetically Chinese Han and have been exposed to coal mine dust for at least 1 year. All study participants used personal protective equipment during working hours. The data also included working history and diagnosed pneumoconiosis. Two sub-queues are established in occupational category (operation, materials handling). Each coal worker, including retired workers, underwent a physical examination every 2 or 3 years. Coal workers are included in our study as long as they started dust exposure from January 1, 1963, to December 31, 2014.

### 2.3. Diagnosis of CWP

According to the Chinese Diagnostic Standard for Pneumoconiosis, the diagnoses are based on occupational history, physical examination, chest radiographs, and pulmonary function tests (National Health and Family Planning Commission of the People’s Republic of China, 2014).

### 2.4. Occupational category

There are some job classifications in the underground mine. It was difficult to separate the operation workers because of the irregular rotation of these 2 jobs among underground coal workers in this mine. Therefore, the miners are sorted into 2 groups according to the mine dust exposure levels and job titles in this study: operation group and materials handling the group.

### 2.5. Dust exposure data

Dust sample was captured twice a month randomly from each monitoring point. The gravimetric and pyrophosphate methods measured the dust concentration and free silica content of each working area. The cumulative dust exposure (CDE) was calculated by multiplying the duration in years by the dust concentration for every coal worker. It is an important index that can be available for each subject


$$\begin{array}{l} CDE=\sum_{j=1}^{n}\left(Cj\times Tj\right) \end{array}$$

In the above formula, n is the job type number of each worker undergoing the observation period; *Cj* is the geometric mean of 8h-TWA (time weighed average concentration) of dust yearly; *Tj* is the observed years. CDE is given in mg·years.

### 2.6. Model introduction

#### 2.6.1. Auto Regression Integrate Moving Average model.

The Auto Regression Integrate Moving Average (ARIMA) model is also called the differential integrated moving average autoregressive model, which is a time series forecasting and analysis method. The model treats the data formed by the research object as a random sequence and excludes individual outliers caused by confounding factors. The entire data set is a random variable dependent on the time factor. This dependence indicates the future development trend of the object. After describing this correlation, the future figure of the time series can be predicted based on this model.

#### 2.6.2. Generalized Autoregressive Conditional Heteroskedasticity model.

The full name of the Generalized Autoregressive Conditional Heteroskedasticity (GARCH) model is the “autoregressive conditional heteroskedasticity model.” It plays a critical role in developing time series variables in econometrics and can fully extract the residual information in the data. The GARCH model belongs to the extension of ARCH, which not only has the advantages of the ARCH model but also can take into account the flat period and the fluctuation period of the time series. Moreover, lower-order GARCH models can represent higher-order ARCH models, making model identification and estimation easier.

### 2.7. Statistical analysis

All data were analyzed using Excel 2007 and SPSS 22.0. *P* < .05 was considered to be statistically significant.

## 3. Results

### 3.1. Baseline characteristics

A total of 8928 coal workers were included in the study. Among them, there were 495 patients (5.54%) with CWP and 8433 coal workers (94.46%) without CWP. For the 495 CWP patients, their average age of onset of CWP was 50.20 ± 11.03 years, their average duration of dust exposure was 26.75 ± 8.51 years, and their average age of first dust exposure was 20.75 ± 4.22years. For the 8433 coal workers without CWP, their average age was 43.82 ± 12.48 years, their average duration of dust exposure was 24.12 ± 10.48 years, and their average age of first dust exposure was 20.07 ± 4.34years. The average dust concentrations in different workplaces decreased with time from Table 1. Among patients with CWP, more than 90% were operation workers, which was significantly higher than that of coal workers without CWP from Table 2. One hundred sixty-seven CWP patients’ (74.89%) and 246 coal workers’ (4.78%) CDE were more than 1000 mg·years. Similarly, 56 CWP patients’ (25.11%) and 4898 coal workers’ (95.22%) CDE were between 100 and 1000 mg·years.

**Table 1:** The average concentrations of dust in different workplaces (mg/m^3^).

Time range	Operation area		Materials handling area	
	Geometric means	Min-max values	Geometric means	Min-max values
<1980	134.9	2.7–1869.6	75.8	1.4–934.8
1980–	115.1	0–1983.3	73.6	45.2–157.7
1990–	42.3	0.1–1730.0	34.3	9.0–84.9
2000–	28.2	0.13–539.5	23	2.6–60.0

**Table 2:** Occupational category and cumulative dust exposure of coal workers.

Variable	Patients with CWP (%)	Coal workers without CWP (%)	*χ* ^2^	*P* value
Job titles				
Operation	449 (90.71)	4323 (51.26)	292.369	<.001
Materials handling	46 (9.29)	4110 (48.74)		
CDE (mg·years)				
≤1000	56 (25.11)	4898 (95.22)	1478.894	<.001
>1000	167 (74.89)	246 (4.78)		

CDE = cumulative dust exposure, CWP = coal worker’s pneumoconiosis.

According to Table 3, we found that there was no statistically significant difference in age and dust exposure years between the materials handling group and the operation group. The number of cases in males was significantly higher than that in females in both groups. The accumulated dust exposure of the materials handling group was significantly higher than that of the operation group.

**Table 3:** Basic characteristics of the research object.

Factor	Materials handling	Operation	*P* value
Age	42.33 ± 10.54	42.25 ± 10.12	.4519
Sex			
Male	4707 (91.31)	5725 (80.16)	
Female	448 (8.69)	1417 (19.84)	<.05
Dust exposure years	24.18 ± 10.37	24.11 ± 10.57	.9745
Accumulated dust exposure	935.8 ± 619.8	883.1 ± 561.6	<.05

### 3.2. Incidence rate of CWP

**Figure 1.**
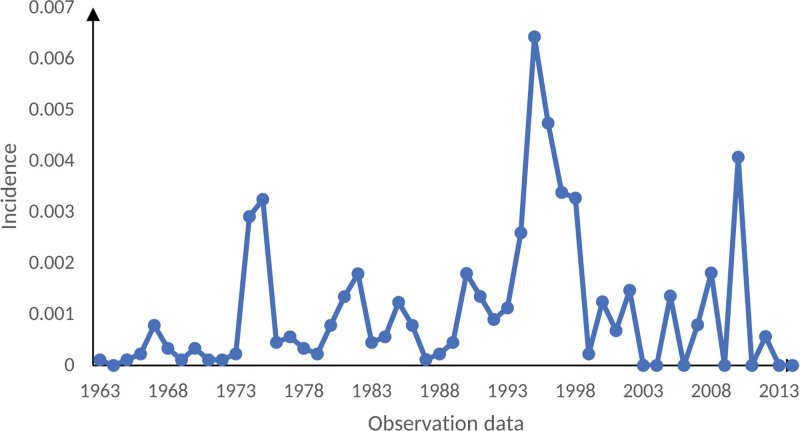
Incidence rate of coal worker pneumoconiosis.

**Figure 2.**
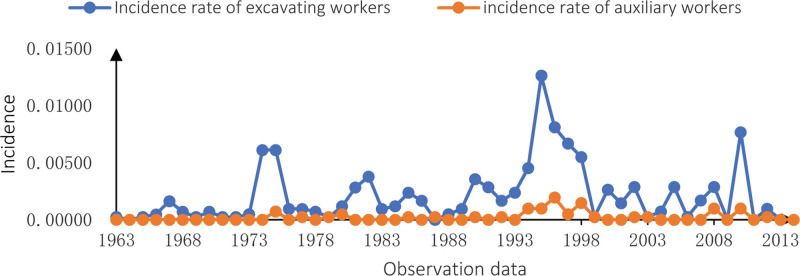
Incidence rate of CWP in different occupation groups. CWP = coal worker’s pneumoconiosis.

In the observations from 1963 to 2014, the annual incidence of mine workers is shown in Figure 1. From 1963 to 1995, the incidence of CWP in the entire mine was on the rise overall, reaching a maximum of 6.43% in 1995. Since then, the incidence of CWP has been at a low level except in 2010, reaching 0 in 2013 and 2014. Figure 2 shows the annual incidence of CWP among tunnel workers, mine workers, transport workers, and help workers. As can be seen from the Table 2, the tunnel and mine workers group had a much higher incidence rate than the transport and helper workers group, with the most considerable difference in CWP incidence between the 2 at 1.16% in 1995.

**Figure 3.**
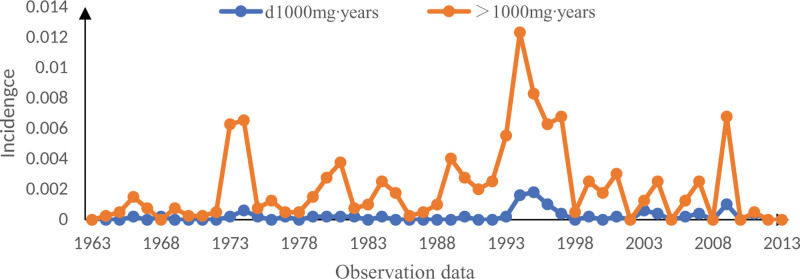
Relationship between different cumulative dust exposure and the incidence of CWP. CWP = coal worker’s pneumoconiosis.

The CDE of all miners is more than 100 mg·year, and with the change in the observation year, the incidence of CWP of the two is gradually at a lower level from Figure 3.

### 3.3. Model establishment

#### 3.3.1. ARIMA model.

**Figure 4.**
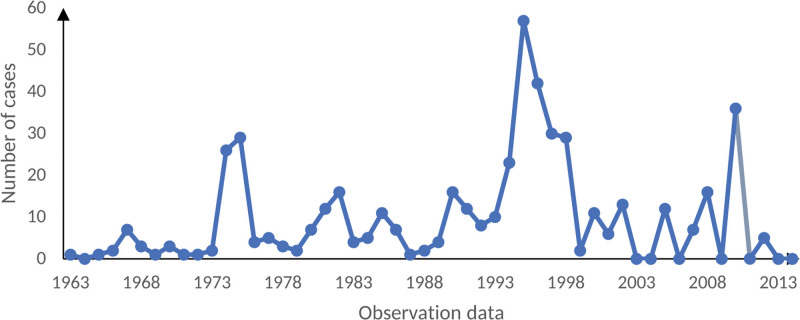
Trend of pneumoconiosis.

It can be seen from Figure 4 that the annual number of patients in the mine is relatively stable without seasonal change. Autocorrelation coefficient function (ACF) diagram and partial correlation coefficient function (PACF) diagram are established.

**Figure 5.**
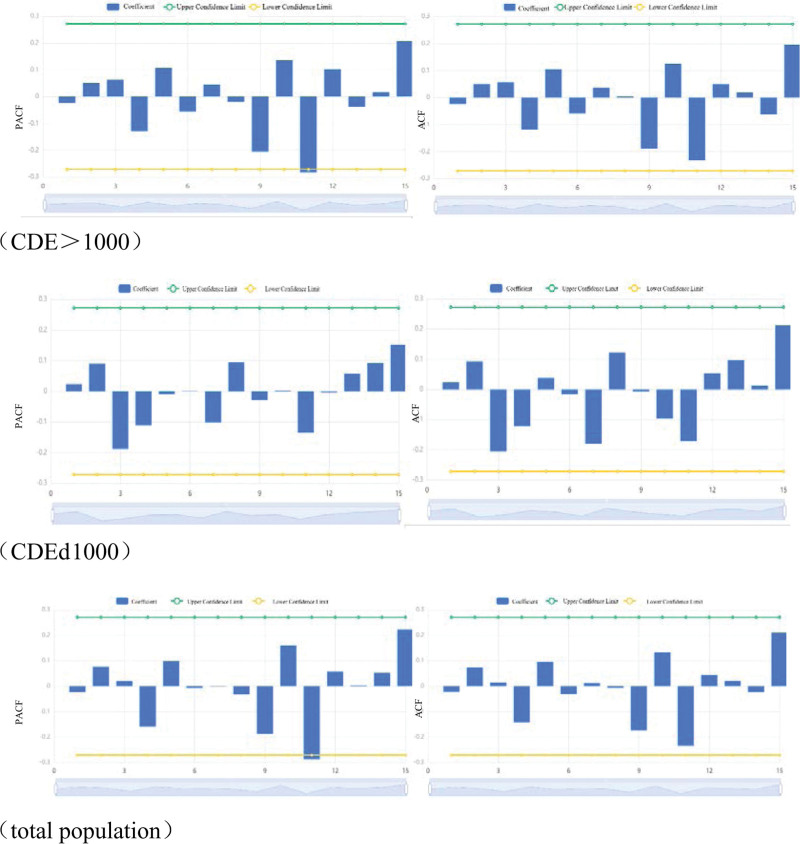
PACF and ACF. ACF = autocorrelation coefficient function, CDE = cumulative dust exposure, PACF = partial correlation coefficient function.

Taking the incidence rate of workers from the mines from 1963 to 2014 as the dependent variable, the ARIMA model is built in the time series module of spss22.0 software. According to the contents shown in Figure 5 (residual ACF and residual PACF diagram), *P* = 1 and *q* = 0 can be obtained. Finally, we determine the annual incidence rate model of the pneumoconiosis in the mine as ARIMA (1, 0, 0) and draw the autocorrelation function (ACF) and partial autocorrelation function (PACF) chart of the residual sequence. The Box-Ljung Q residual statistics are meaningless (*P* = .939), indicating that the residual sequence is white noise and fitted *R*^2^ = 0.404, so we can get that using ARIMA (1, 0, 0), the model predicts the future incidence rate of the mine is reasonable.

#### 3.3.2. Generalized autoregressive conditional heteroskedasticity.

**Figure 6.**
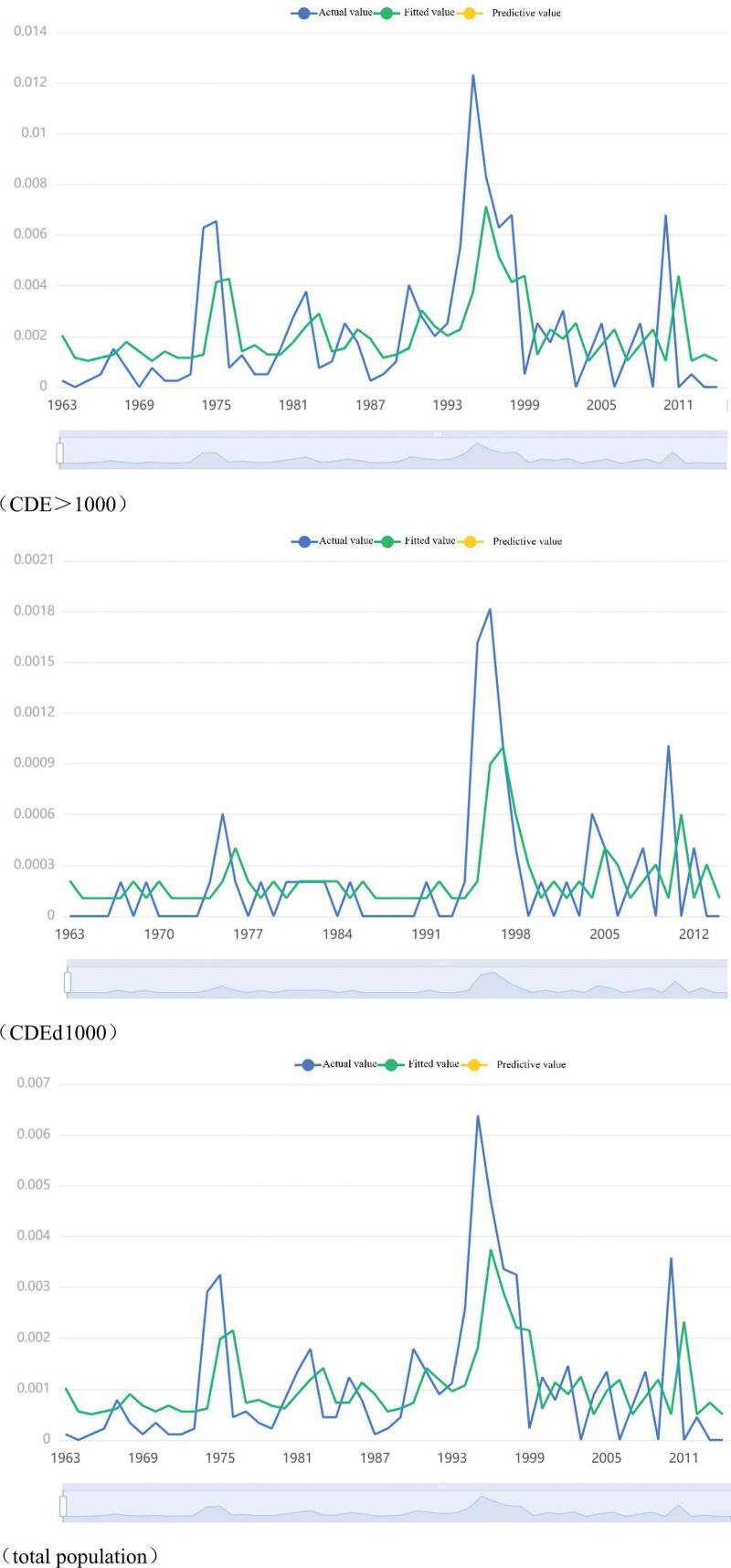
Time series results plot. CDE = cumulative dust exposure.

Three kinds of result volatility graphs were obtained by taking the same population as the research object and building the model with the same dependent variable, as shown in Figure 6.

### 3.4. Evaluation of model prediction effect

The model prediction effect evaluation indicators selected this time include Akaike information criterion and *R*^2^, of which *R*^2^ can better reflect the fit between the prediction and the actual value. From Table 4, we found that the Akaike information criterion value of the GARCH model is stable, and the *R*^2^ is relatively large, where *R*^2^ (total population) = 0.532.

**Table 4:** Model prediction effect evaluation.

Model	Grouped	AIC	*R*²
ARIMA (1,0,0)	CDE ≤ 1000	−680.043	0.245
	CDE > 1000	−480.845	0.246
	Total	−551.154	0.26
GARCH	CDE ≤ 1000	−12.89	0.23
	CDE > 1000	−8.859	0.485
	Total	−10.24	0.532

AIC = Akaike information criterion, ARIMA = Auto Regression Integrate Moving Average, CDE = cumulative dust exposure, GARCH = Generalized Autoregressive Conditional Heteroskedasticity.

## 4. Discussion

Coal remains the primary energy resource in China, and coal dust exposure is one of the essential hazards during coal mine. Although the dust concentration and incidence of pneumoconiosis decreased markedly in the past decades, dust exposure in the industrial environment has not been fully controlled.^[14,15]^ According to a cost-effectiveness study in China, the economic loss ratio of investment to recovery is 1:1.43.^[16]^ CWP is also one of the occupational diseases that has been most studied, but there is no effective treatment on the market. Disease prediction is a critical step in transforming from passive prevention to active prevention and can help establish a complete prevention strategy. While, as a kind of severe pulmonary disease, the incidence of CWP can be affected by many factors, making it difficult to predict the trends accurately.^[17,18]^ Many prediction methods estimate and predict the incidence of pneumoconiosis and related diseases. Tan et al showed that a Grey GM (1, 1) model met the requirements of model predictions and can be applied to estimate new cases in the next three years.^[19,20]^ A logistic regression model was used to predict the prevalence of pleural plaques in the workers exposed to asbestos in France, and a 0.8% to 2.4% yearly increase was reported for a mean exposure of 1 f/mL.^[21]^ Tse et al established a unique score system with an excellent internal validity to assess the risk for silicosis, identify high-risk workers, and provide scientific guidance for clinical decision-making. Therefore, developing the corresponding prediction model by the epidemiological characteristics of different exposure populations should be essential to disease prevention. Notably, CWP is impacted by many risk factors other than the dust particle inhalation’s quality, character, and response time.

The ARIMA model can incorporate the mixed effects of many complex factors into time variables and comprehensively consider factors such as periodicity, long-term trends, and random fluctuations. This is a prominent advantage of time series analysis in disease prediction.^[22]^ The previous study performed a fitting analysis on various models and predicted the incidence of syphilis in Gansu Province based on each model.^[23]^ The GARCH model is a generalization of the ARCH model proposed by Bollerslev and can be used to deal with data with an extensive fluctuation range.^[24]^ The emergence and expansion of the model provide important means for both probability and prediction.

This article uses the residual ACF and residual PACF diagrams in the time series model in the SPSS software to determine the *P* and *q* values of the model. Then builds a GARECH model and compares the prediction effect with ARIMA. By comparing the relevant predictive standard indicators, we found that the GARCH model is more suitable for the population of this study. Based on the above forecast results, we believe that the occupational hazards caused by dust are still worthy of our attention. Each coal worker should also receive regular physical examination according to the “Guideline of Occupational Health Surveillance (GBZ188-2014).” Besides, applying more advanced and automated coal mine equipment is the best way to reduce the concentration of mine dust.

There are also some shortcomings in this article, especially when analyzing without considering different covariates, except for CDE, such as socioeconomic status, health status, etc. In future research, we will gradually improve.

## 5. Conclusion

This study analyzed epidemiological data from 1963 to 2014 on nearly 9000 Chinese coal miners to forecast trends in CWP incidence. GARCH and ARIMA time series models were used. GARCH more precisely modeled historical volatility in CWP rates over time versus ARIMA. While overall incidence declined with dust reductions, risk levels remained elevated. Both models struggled to accurately predict short-term trends, highlighting the importance of sustained occupational surveillance coupled with improved exposure tracking to better protect workers against preventable respiratory disease.

## Acknowledgments

We thank the research subjects for their active participation and the strong support of Jiangsu Center for Disease Prevention and Control, as well as the teachers and students who provided help for the data processing of this paper. Finally, the authors thank the Jiangsu Science and Education Strong Health Innovation team for its funding.

## Author contributions

**Writing—review & editing:** Peng Sun.

**Methodology:** Bosheng Wang.

**Resources:** Lei Han, Baoli Zhu.

**Supervision:** Lei Han.

**Conceptualization:** Hengdong Zhang, Ming Xu.
